# Closed-loop SCS and sensing: now and in the future

**DOI:** 10.1093/pm/pnag039

**Published:** 2026-06-10

**Authors:** Krishnan Chakravarthy, Vwaire Orhurhu, Jan Willem Kallewaard, Leonardo Kapural, Alaa Abd-Elsayed, Yashar Eshraghi, Lakshmi Rekha Narra, Priyanka Ghosh, Samir Sheth, David W Lee, Rajiv Reddy, Lisa M Johanek, Julia E Gamache, David A Dinsmoor, AnneMarie K Brinda

**Affiliations:** VA San Diego Healthcare, San Diego, CA, United States; Innovative Pain Treatment Solutions and Surgery Centers, San Diego, CA, United States; Solaris Research Institute, San Diego, CA, United States; University of Pittsburgh Medical Center, Williamsport, PA, United States; University of Pittsburgh Medical Center, Pittsburgh, PA, United States; Rijnstate Hospital, Department of Anesthesiology and Pain Management, Arnhem, Netherlands; Amsterdam University Medical Center, Amsterdam, Netherlands; Carolinas Pain Institute, Winston-Salem, NC, United States; University of Wisconsin, Department of Anesthesia, Madison, WI, United States; Ochsner Health System, Louisiana State University School of Medicine, New Orleans, LA, United States; Rutgers Robert Wood Johnson Medical School and Rutgers Cancer Institute of New Jersey, New Brunswick, NJ, United States; Remedy Medical Group, San Francisco, CA, United States; Sutter Health, Roseville, CA, United States; Fullerton Orthopedic, University of California, Irvine, Fullerton, CA, United States; Department of Anesthesiology and Pain Medicine, University of California San Diego Health Sciences, San Diego, CA, United States; Medtronic Neuromodulation, Minneapolis, MN, United States; Medtronic Neuromodulation, Minneapolis, MN, United States; Medtronic Neuromodulation, Minneapolis, MN, United States; Medtronic Neuromodulation, Minneapolis, MN, United States

**Keywords:** spinal cord stimulation, spinal cord, closed loop SCS, evoked compound action potential

## Abstract

**Background:**

Spinal cord stimulation (SCS) for chronic pain has undergone many advancements over the years, including the introduction of novel stimulation waveforms, expansion to treat new pain conditions, and the more recent implementation of evoked compound action potential (ECAP) controlled, closed-loop (CL) SCS. The ECAP—a measure of neural activation—may be used as a biosignal for adjusting stimulation amplitudes to avoid under- and overstimulation associated with spinal cord movement relative to the SCS lead.

**Content overview:**

Using ECAPs in a CL system relies on successfully isolating the neural response signal from the relatively large stimulation artifact. A study with the first commercialized CL-SCS device investigated pain relief with low-frequency stimulation within a therapy window described as ranging from initial perception of sensation to discomfort (paresthesia window). In contrast, another commercial system with CL technology includes additional functionality to support consistent therapy with higher frequency stimulation set at or near the perception threshold. This more recently approved CL-SCS system leverages advanced artifact suppression and flexible CL control algorithms to afford dose control for conventional, high-frequency (up to 1200 Hz), and contemporary SCS waveforms.

**Conclusions:**

Here, we discuss the history and motivation for CL systems in SCS and the challenges faced when implementing such technology.

## The dynamic movement of the spinal cord

**Figure 1:**
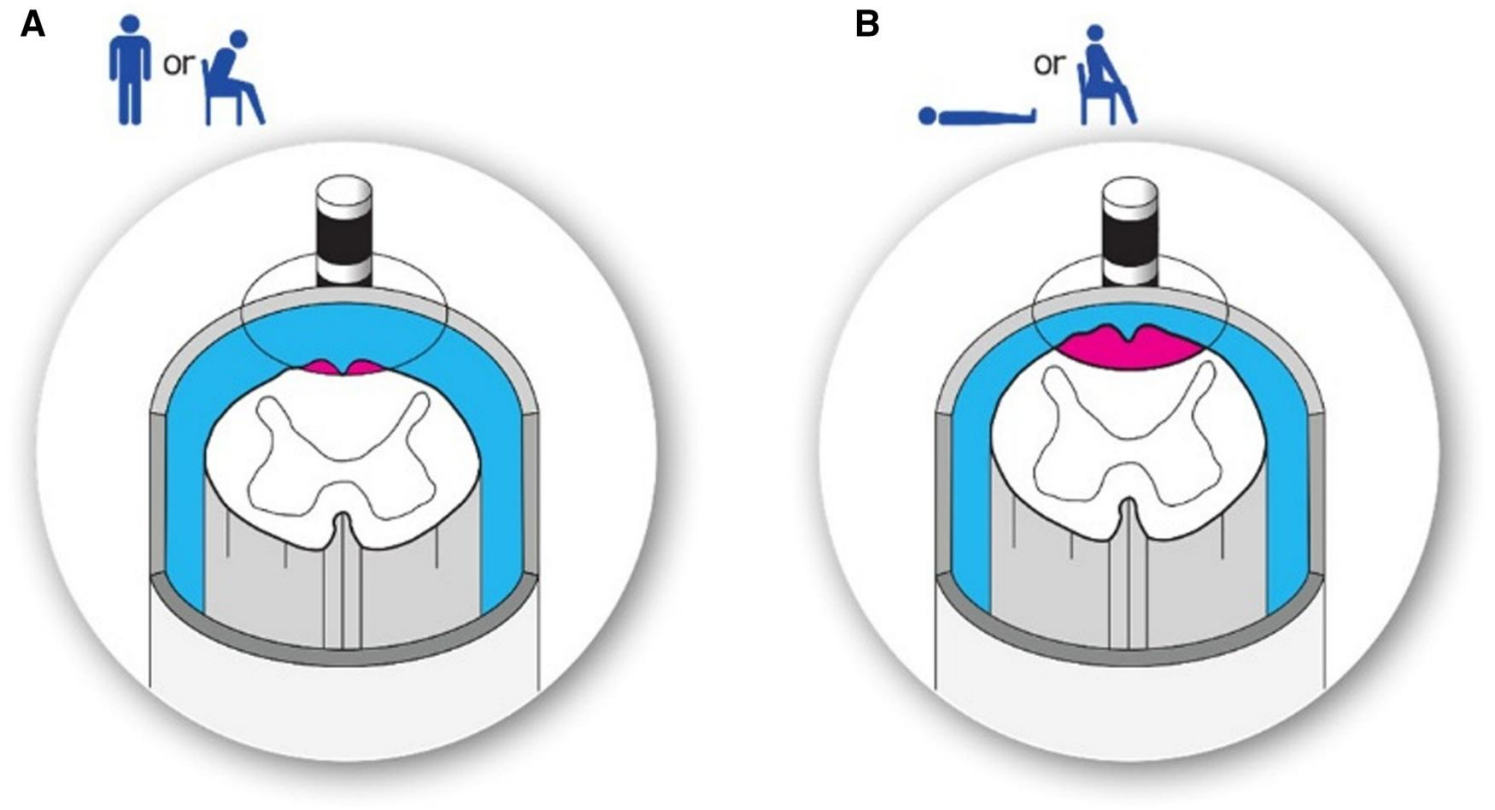
Variation in the volume of tissue activation (VTA) with cord motion in spinal cord stimulation (SCS) patients. (A) When the spinal cord is further from the SCS leads, the CSF (blue) separating the spinal cord and the lead increases, while the VTA (pink) in the spinal cord decreases. Changes in VTA can occur with all SCS systems, limited to fixed parameter stimulation during changes in position and other daily activities such as laughing or coughing. This decrease in VTA may be caused by standing or sitting in an upright, slouched position. (B) When the distance between the lead and the spinal cord decreases, the VTA in the spinal cord increases. This change in VTA may be caused by lying in the supine position or arching the back. This increase in VTA is often referred to as overstimulation and may be felt by the patient as an uncomfortable onset or increase in paresthesia sensation.

Spinal cord stimulation (SCS) is a proven therapy for treating chronic pain refractory to conventional medical management.^1^ Electrical pulses to the dorsal spinal cord may relieve pain by modulating the transmission or perception of painful signals. Unlike stimulation therapies with electrodes in direct contact with the target tissue (eg, cardiac pacing and deep brain stimulation), SCS leads are positioned epidurally, leaving the dura mater and a layer of cerebrospinal fluid (CSF) between the electrodes and their target. Although the lead stabilizes in scar tissue over time, a well-known challenge is the consistent delivery of SCS therapy due to spinal cord movement relative to the lead (Figure 1). This movement causes variation in the volume of tissue activation (VTA) that occurs with each stimulation pulse. This VTA variability may result in uncomfortable stimulation, leading to manual therapy adjustments and changes in patient compliance, such as reducing stimulation amplitude or eliminating device use. Reducing the stimulation amplitude may lead to suboptimal energy delivery. Therefore, addressing this issue is crucial to ensure the consistent and durable effectiveness of all types of SCS therapy.

The factors that impact the spatial relationship between the spinal cord and the location of an SCS lead on the dural surface primarily include gravity and spinal flexion/extension (Figure 1). Gravity moves the cord closer to the dura mater and leads when supine. Magnetic resonance imaging (MRI) shows that, when changing position from supine to prone, the cord-to-dura distance increases by an average of 2.2 mm at T11 and 3.4 mm at T12.^2^ Spinal flexion causes the largest number of deformations in the nervous system. Flexion and extension can also result in unexpected cord deformation at specific spinal cord levels. For example, in a cadaveric study of the cervical spine, the spinal cord rests against the dorsal spinal canal during extension in the supine position, and flexion raises the cord ventrally.^3^ Position and spinal movement alter the SCS lead-cord relationship, subsequently changing the VTA.

The dynamic nature of the spinal cord introduces a challenge in SCS therapy, as the target for electrical impulses undergoes continuous movement. While the lead remains stable relative to the dura, the spinal cord is moving dynamically throughout activities of daily living. Multiple studies have reported the association between SCS perception threshold and posture, with a lower perception threshold when in a supine position relative to a standing one.^4–9^ Further, patients may feel increased stimulation during sudden movements such as sneezes, coughs, or stretches. The spinal cord moves closer to the SCS lead during these movements, resulting in increased neural activation.^10^ The patient experience can be affected by a constantly changing neural activation due to large and small movements encountered in their everyday life.

## The need for consistent SCS therapy

Historically, the patient has been responsible for managing changes in therapy to avoid uncomfortable overstimulation events by making proactive or reactive adjustments with their patient programmer. If the stimulation adjustments become burdensome over time, the patient may prevent overstimulation by indefinitely reducing the stimulation amplitude and maintaining lower-than-effective stimulation. Therefore, another consequence of spinal cord movement may be that the patient settles for a “subtherapeutic dose” of stimulation (Box 1). This patient-triggered understimulation may have the long-term impact of reduced pain relief which in turn may result in abandonment of the therapy. Avoiding uncomfortable paresthesia while maintaining an effective stimulation dose could be a key advancement towards preventing treatment loss in SCS patients.

Box 1. Stimulation as a “dose”

Both conventional low-frequency SCS and more recently developed subthreshold programming methods have been described with analogies to the concept of a pharmacologic dose of a drug. Electrical charge per unit of time, time with therapy On, and whether therapy is delivered in an ideal range have represented the active compound of the therapeutic dose. The concept of electrical charge as an active compound is useful for describing the range between an ineffective therapy amount and an amount causing treatment-limiting side effects, unique to each patient. Variables of frequency, pulse duration, amplitude, and duty-cycle may be adjusted to find the correct therapy settings, or dose, for an individual patient. No linear dose-response relationship between representations of SCS dose and pain relief has been demonstrated as predictive of therapy outcomes. However, ensuring consistent SCS dosing reduces the important variable of therapy compliance, whether it deviates from ideal based on intentional patient modifications or unintentional spinal cord motion. In this article, the term “dose” refers to the amount of electrical charge *received by the spinal cord* rather than the amount *delivered by the device,* because those often differ in SCS, given the ever-changing distance between the spinal cord and the device. Additionally, the concept of SCS dosing is, of course not limited to the paresthesia-inducing, continuous Aβ fiber activation characteristic of conventional, low-frequency SCS.

The risk of unwanted or unanticipated changes in stimulation and the impact on the patient are apparent when using conventional, paresthesia-based SCS therapy. One strategy to mitigate transient paresthesia-centric overstimulation involves the application of subperception SCS modalities. Typically, subperception therapy is achieved by setting the stimulation amplitude at 50% to 70% of the perception threshold, creating a safety margin that prevents patients from feeling the stimulation as they move. SCS using 10 kHz electrical stimulation was FDA approved in 2015 as a subperception therapy for pain relief for patients with chronic back and leg pain.^11^^,^^12^ The following year, a specific type of burst programming, BurstDR SCS, was FDA-approved.^13^ BurstDR uses pulse trains of around 500 Hz with an interburst frequency of 40 Hz. While not designated as a “paresthesia-free” therapy, BurstDR is typically programmed to subperception therapy amplitudes.^14^ Subperception programming with frequencies up to 1200 Hz was efficacious in the WHISPER study, indicating that ultra-high frequencies may not be required for adequate subperception pain relief.^15^ Similarly, FAST programming, which stimulates at a frequency of 90 Hz at a level ideally at or near the perception threshold, was also found to be effective.^16^

For patients experiencing adequate benefit from SCS delivered at stimulation amplitudes below the sensory threshold, the impact of spinal cord movement may not be noticeable. While the sensation may not change, the dose of stimulation the spinal cord receives will still vary between 30% to 80% with position changes.^4–9^ Additionally, fast transient spinal cord movements may result in perceptible neural activation, especially when high frequencies and wide pulse widths are used. Studies show that as frequency increases (with a constant pulse width), the threshold for neural activation decreases. For example, Abejon et al^17^ found that the discomfort threshold decreased from 9.20 mA at 40 Hz to 1.85 mA at 1200 Hz. Similarly, increasing the pulse width from 200 to 1000 μs decreased the discomfort threshold from 11.62 mA to 5.74 mA, respectively.^18^ Therefore, patients programmed to higher frequencies or wider pulse widths may have a smaller subthreshold “buffer” before hitting perception and discomfort thresholds. Therapies with high rates and short pulse widths (ie, 10 kHz and 30 μs) have been reported to cause unpleasant sensations described as “tightness” and “pressure” when close to activation threshold.^19^

A survey of 100 patients across multiple device manufacturers—Medtronic (33%), Nevro (28%), Boston Scientific (24%), and Abbott (15%)—examined the frequency of unwanted sensations in those programmed to subperception therapy.^20^ Among these patients, 80% used subperception therapy; within this population, 59% reported that stimulation could feel too strong during certain activities or body positions. Additionally, 70% of all patients adjusted their stimulation proactively to manage sensation changes, and 85% expressed interest in a self-adjusting solution that avoids shocks or jolts. Despite assumptions that subperception therapy prevents stimulation issues, this survey highlights the ongoing need for patients to manage over- and understimulation in their SCS therapy. While therapies with little or no paresthesia sensation while stationary may seem appropriate to this challenge, they do not solve the dose consistency problem because spinal cord movement is still not addressed.

## Closed-loop solutions

Despite advancements in SCS therapy, most systems are fixed-output, only changing stimulation parameters when the patient or clinician makes therapy adjustments. These open-loop (OL) systems have no automatic feedback mechanism. They can be challenging to optimize and maintain a preferred stimulation delivery level, requiring iterative adjustments using patient feedback. Once a stimulation amplitude is set in the clinic, the patient may still need to make manual adjustments at home as they change positions and engage in daily activities. In contrast, CL systems are controlled in real-time with automatic adjustments based on detected signals, allowing the desired stimulation level, or dose, to be maintained once the patient returns home.

The feedback signal in a CL system is variable and dependent on the clinical context, including the disease state and how the signal is interpreted and used in the therapeutic framework. For example, rate-responsive cardiac pacing utilizes input signals to determine the need for a faster heart rate.^21^ Body movement is one such input, and accelerometers or piezoelectric crystals have been incorporated into pacemakers as practical tools for detecting movement and adjusting the pacing rate accordingly. Additionally, parameters directly associated with cardiac function, such as the QT interval or impedance changes reflecting heart muscle contractility, can serve as feedback signals. These advancements in cardiac pacing demonstrate the integration of signal sensing with closed-loop systems to adjust the heart rate dynamically based on physiological needs.^21^ Similarly, CL neuromodulation therapy may be implemented in various ways depending on the input signal. In deep brain stimulation (DBS) therapy, stimulation amplitude is adjusted based on detecting signals specific to the state of Parkinson’s disease.^22^ The CL feedback signal used in DBS for Parkinson’s disease is known as a local field potential (LFP).^23^ When observed in the beta frequency range, this naturally occurring signal is closely associated with symptoms, medication effects, and DBS stimulation amplitude. Higher beta LFP amplitudes are correlated with more severe motor symptoms, prompting the CL algorithm to increase stimulation. Conversely, when beta LFP amplitudes are lower, the algorithm reduces stimulation accordingly. While DBS CL systems can modulate stimulation based on signals related to the disease state, SCS CL algorithms focus on therapy adjustment to address the dose consistency issue described in the previous section.

## Posture-responsive stimulation using accelerometer technology

To address spinal cord movement during posture changes, the first adaptive SCS solution used an embedded 3-axis accelerometer in the implanted neurostimulator to track patient position. Still available in Medtronic SCS systems, it detects positions (reclining, upright, supine, prone, and side-lying) and automatically adjusts stimulation using preset amplitudes. This technology was the first in SCS to use an objective signal to deliver patient-preferred stimulation based on body position.

The Restore Sensor Study demonstrated the benefits of automatically adjusting therapy with posture-responsive technology in SCS.^24^ The study, involving 76 participants, showed that 86.5% experienced improved pain relief or convenience, 88.7% reported better pain relief with posture-responsive stimulation compared to manual adjustments, and 90.1% intended to continue using the technology. Real-world studies also confirmed better patient-reported outcomes with adaptive stimulation.^25^^,^^26^ Overall, automatic adjustment based on position changes provides significant benefits for patients.

While effective at automatically adjusting stimulation based on body position, the technology did not address all issues related to spinal cord movement relative to the SCS lead. The accelerometer is insensitive to non-posture-related spinal cord movements, such as coughing, laughing, breathing, and extension/flexion movements. Another limitation of relying on body position detected by this technology is that the time scale of adjustment is within the range of seconds. At the same time, stimulation variations occur in the sub-second range. More effective solutions that predict the stimulation current received by the spinal cord are needed to ensure the stimulation dose is timely controlled during all movements of daily life.

## Sensing the evoked compound action potential (ECAP)

**Figure 2:**
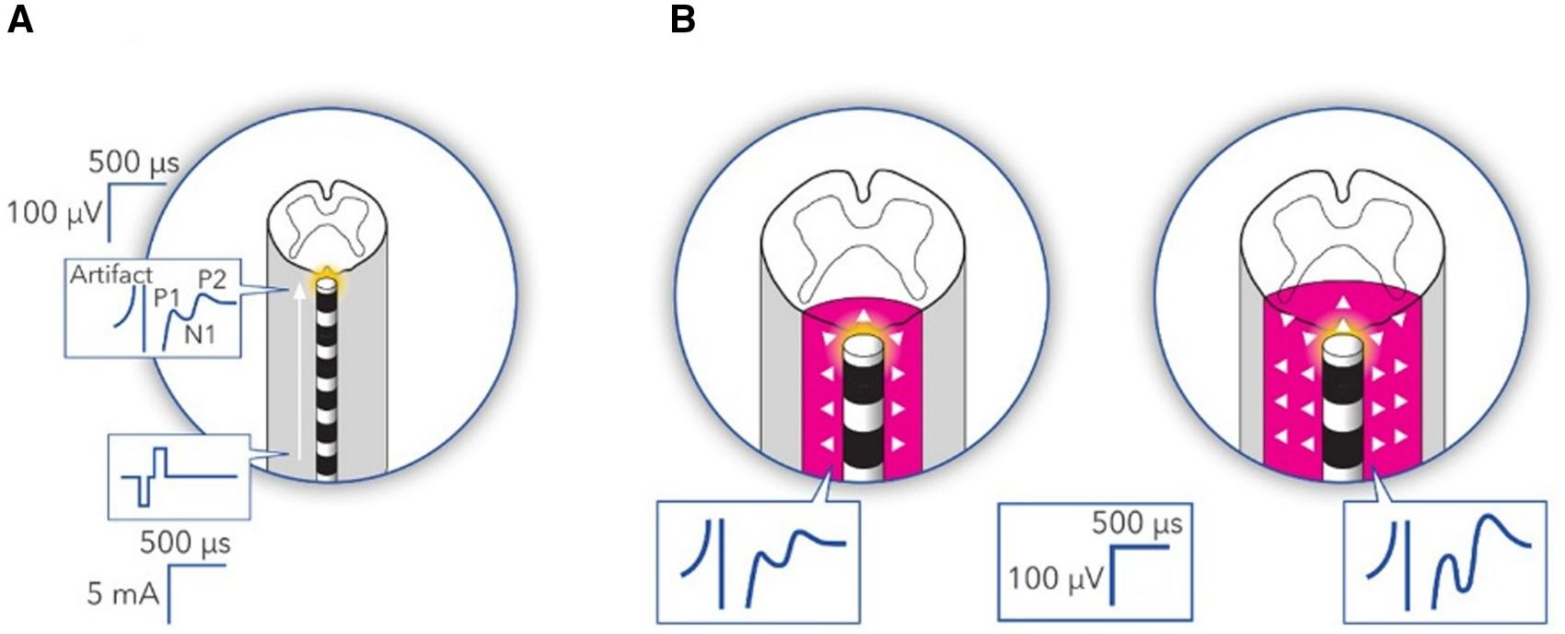
Recording the spinal ECAP. (A) The spinal ECAP waveform generally consists of a first peak (P1), a trough (N1), and a second peak (P2). ECAPs generated by stimulation at one end of an SCS lead can be recorded from distal contacts on the same lead. (B) In OL systems with fixed-output stimulation, the volume of tissue activated (VTA) changes during daily living activities. The ECAP provides a measure of the VTA—smaller ECAPs represent less neuronal activation; larger ECAPs represent higher levels of neural activation.

In addition to accelerometer signals, it has become possible to sense bioelectrical signals at the SCS lead electrodes. These biosignals, known as evoked compound action potentials (ECAPs), relate to activation of a population of neurons in the dorsal columns of the spinal cord, primarily comprised of Aβ fibers conducting non-painful touch stimuli.^27^ An ECAP occurs when electrical stimulation causes compound, synchronous activation of multiple Aβ fibers in the dorsal column of the spinal cord, thus representing a summation of the activity of numerous neurons. The spinal ECAP waveform generally consists of a first peak (P1), a trough (N1), and a second peak (P2) (Figure 2A). As stimulation increases or the spinal cord moves closer to the SCS lead, the ECAP amplitude (a positive number calculated as the voltage difference between P2 and N1) and the VTA increase (Figure 2B). Incorporating the ECAP amplitude as a control signal for a CL SCS system provides a means to compensate for variability in the VTA by automatically controlling stimulation amplitude of the SCS device.

Although a novel technique in SCS therapy, the use of ECAPs has been widely studied in the auditory field for several decades.^28^ Implementing ECAP technology has been a key advancement in cochlear implants (CI), which target the inner ear to improve hearing in people with auditory impairment. Historically, when fitting a CI, the behavioral stimulation threshold and maximum comfortable hearing levels for each electrode contact were manually preset in the clinic. However, these stimulation levels have been shown to change throughout the life cycle of an implant.^29–32^ Therefore, it was necessary to manually adjust the CI periodically. To restore speech perception today, the settings of the CI are optimized for the individual patient using auditory nerve ECAPs. The auditory nerve ECAP represents the neural response of spiral ganglion cells lining the inner part of the cochlea. ECAPs are induced by stimulation on multiple CI electrodes, and the information is used to assist in creating a patient-specific map of each electrode’s lower and upper stimulation levels, which enables efficient optimization of the CI settings.^31^^,^^32^ Given the successful utilization of ECAPs in CI technology, engineers saw this as an opportunity to innovate by incorporating ECAPs into other neuromodulation technologies, namely SCS.

## Sensing in the spinal cord: Overcoming challenges

Despite the promise of ECAPs, the feasibility of leveraging their use for sensing in SCS poses several challenges. A recent report described how changes in pulse width, electrode charge configuration, and spacing between stimulation and sense electrodes can dramatically impact the fidelity of the ECAP recording.^33^ Any change in these parameters can make the relatively small ECAP more challenging to distinguish from the concurrent stimulation artifact, or the electrical signal originating from the stimulating electrode rather than the Aβ fibers.

Another issue is the variability of the distance between the electrodes and the neural target, which is linked to the CSF layer’s thickness.^33^ The shunting effect caused by the CSF layer, which is conductive and can disperse the electrical current from the lead, reduces the amplitude of recorded spinal ECAPs. This adds difficulty to distinguishing the ECAP from the stimulation artifact.^34^ Therefore, the chosen artifact reduction method must work with high precision and accuracy to capture the accurate neural response, and misclassification of artifacts as neural signals is limited. A template correlation method estimates the ECAP by correlating the measured signal with an artificial ECAP signal. However, this method may result in under- or over-estimating the ECAP signal, which is a concern when implemented in a conventional paresthesia-based CL-SCS system.^34^ Conversely, using a differentiator or high-pass filter technique results in a higher neural response-to-artifact output while remaining computationally efficient.^34^ The chosen artifact subtraction method impacts how well a CL algorithm can perform, particularly when sensing ECAPs close to the perception threshold.

Experience gained from recording small signals while avoiding stimulation artifacts in other therapies, such as DBS, is now being applied to sensing small neural signals in the spinal cord. Sensing LFPs in DBS therapy began with research using investigational devices, which could record electrophysiological signals from DBS leads.^35^^,^^36^ Sensing and adaptive CL technology is now possible in commercial neurostimulators, the first DBS devices capable of chronic, at-home sensing. Significant technical advancements were required to enable a stimulation device capable of sensing these small neural signals.^37^

## Closed-loop with conventional SCS

**Figure 3:**
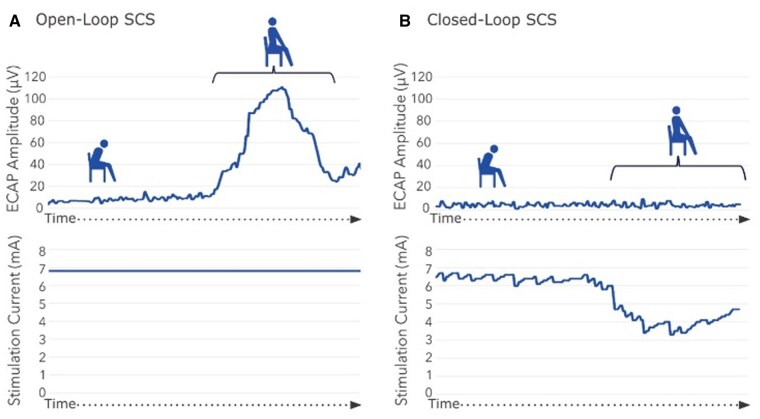
ECAPs recorded during SCS therapy delivered in an OL configuration (A) or a CL configuration (B). The stimulation is set at approximately 7 mA in this specific OL example. (A) When the patient performs a back arch, the spinal cord moves closer to the lead, causing an increase in VTA. The increased ECAP amplitude indicates an increase in VTA. (B) In contrast, CL-SCS controls the stimulation amplitude to ensure a more consistent VTA when a back arch is performed; when an increasing ECAP signal is detected, the therapy amplitude is instantly decreased.

Currently, 2 SCS manufacturers have commercially available ECAP-enabled CL-SCS systems. The foundation of CL approaches in these devices is to sense an ECAP signal elicited by a relatively low-frequency input signal (eg, 10-100 Hz). In contrast to an OL system in which stimulation amplitude is fixed, the CL system adjusts stimulation amplitude to maintain a more consistent ECAP amplitude (Figure 3). After each stimulation pulse, the resulting amplitude of the ECAP is used to determine the stimulation amplitude of the next pulse. If the ECAP amplitude is of an appropriate size, based on the desired therapy for the patient, no change is made to the therapy. A low-amplitude ECAP triggers an increase in therapy amplitude, while a high-amplitude ECAP triggers a rapid reduction in therapy amplitude.

The use of ECAP-enabled closed-loop SCS for delivering low-frequency therapy near or above paresthesia level has been studied using the Evoke system (Saluda Medical). In the EVOKE randomized controlled trial (RCT), the therapeutic window was characterized by the range of ECAP amplitudes—from the lowest level at which patients first perceived stimulation, up to the highest level the patient could tolerate.^38^ The EVOKE RCT demonstrated the advantages of an ECAP-based CL feature over conventional fixed-output SCS with paresthesia-centric therapy.^38^ This study randomized 134 subjects to either CL-SCS (*n* = 67) or OL-SCS (*n* = 67) and followed both arms for 2 years. At the 2-year follow-up, the responder rate for overall pain in the CL arm was 79.1% compared to 53.7% in the OL arm.^39^ Subjects then had the option to crossover to the other treatment; 40 subjects crossed from OL to CL, and 80% of these selected to continue using CL-SCS.^40^ At the 3-year follow-up, data were collected from subjects completing the study in CL SCS (*n* = 62) and OL SCS (*n* = 24).^40^ The responder rate for overall pain was 77.6% for subjects using CL SCS and 49.3% for subjects using OL SCS. On the Patient Global Impression of Change (PGIC), 81.0% of subjects on CL SCS and 66.0% of subjects on OL SCS improved, feeling Very Much Improved or Much Improved, respectively. This is the only RCT conducted to demonstrate that ECAP-based CL programming provides better pain relief than OL programming. Regarding safety, nonserious study-related adverse events were more frequently reported in the CL arm compared to reports in the OL arm at both the 1-year (CL-SCS: 22, OL-SCS: 9)^38^ and 2-year follow-up (CL-SCS: 28, OL-SCS: 14).^39^ However, there was no statistical difference in the safety profile between the 2 treatments (difference in rate of study-related adverse events: 6.0 [95%CI, −7.8 to 19.7]).^39^

The EVOKE RCT studied a CL algorithm developed to maintain a level of paresthesia by controlling stimulation amplitude to keep the ECAP amplitude within a prescribed range, described as a “therapeutic window.” Subjects in the CL and OL/fixed-output arms were programmed to low-frequency therapy (mean, approximately 40.0 Hz; range, 10.0-80.0 Hz).^38^ Subjects in the OL-SCS and CL-SCS arms used their therapy similarly (> 80% time used). However, at 12 months, the CL-SCS subjects maintained a higher therapy amplitude and remained within their programmed paresthesia window settings 95.2% of the time. In contrast, subjects in the OL-SCS arm tended to turn therapy down and were only *within* the intended amplitude range 47.9% of the time at 12 months. Further, OL-SCS subjects were *below* the targeted therapy level 48.3% of the time and rarely *above* the intended amplitude range. These data exemplify the behavior of patients with fixed-output, OL therapy—they tend to turn the therapy down to avoid overstimulation. In contrast, low-frequency therapy with CL control is kept in the patient-specific ECAP amplitude window. The data suggest that subjects stayed in their paresthesia window because the CL algorithm prevented overstimulation, and that avoiding overstimulation is key to preventing patient-controlled understimulation.^38^

**Figure 4:**
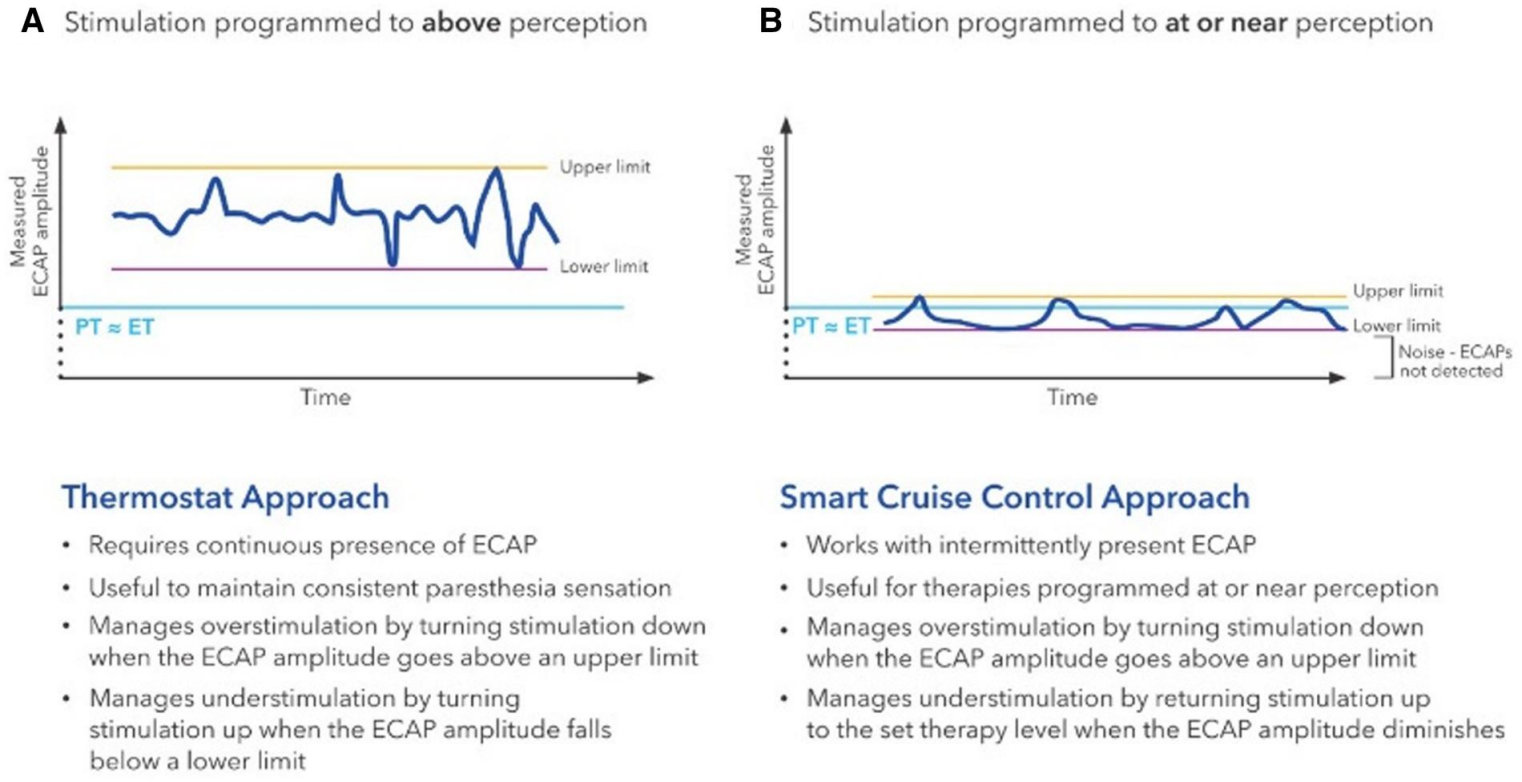
Visualization and explanation of the different CL-SCS programming approaches. (A) When stimulation is programmed above the perception threshold, CL-SCS maintains the stimulation amplitude within an ECAP-inducing window. This can be considered a “thermostat” approach focused on a targeted ECAP amplitude. (B) The “smart cruise control” approach constrains stimulation amplitude at or below the perception threshold. This strategy focuses on staying as close to the perception threshold as possible while preventing the presence of an ECAP and, therefore, limiting paresthesia.

The CL algorithm in the Inceptiv device is programmable and patient-specific. For patients who prefer to feel comfortable stimulation sensation (paresthesia) during stimulation, the CL algorithm works similarly to the aforementioned approach by adjusting the stimulation amplitude to tightly maintain the sensed ECAP amplitude between an upper and a lower limit, or ±1.5 µV of a specific ECAP target, in other words, a thermostat-like approach. The physician and patient determine these limits as comfortable and therapeutic (Figure 4A). At a continuous rate of 50 Hz, the system measures the elicited ECAP amplitude and determines whether to increase, decrease, or hold the stimulation amplitude. If the ECAP amplitude exceeds the upper limit, the stimulation amplitude is decremented until the ECAP amplitude is less than the upper limit. If the sensed ECAP amplitude falls below the lower limit, the stimulation amplitude is incremented until the ECAP amplitude rises back above its lower limit. The rates at which the stimulation amplitude increases/decreases are also programmable. This way, the therapy dose received—as measured by the ECAP amplitude—consistently remains within a range specified for each patient, similar to setting a target temperature on a thermostat controlling both heating and cooling inside a home or automobile. This approach has been studied with paresthesia-centric CL-SCS, where the CL-SCS is configured to approximate an ECAP amplitude of 1.4 times the ECAP threshold.^41^

## Developing closed-loop therapy for multiple waveforms

**Figure 5:**
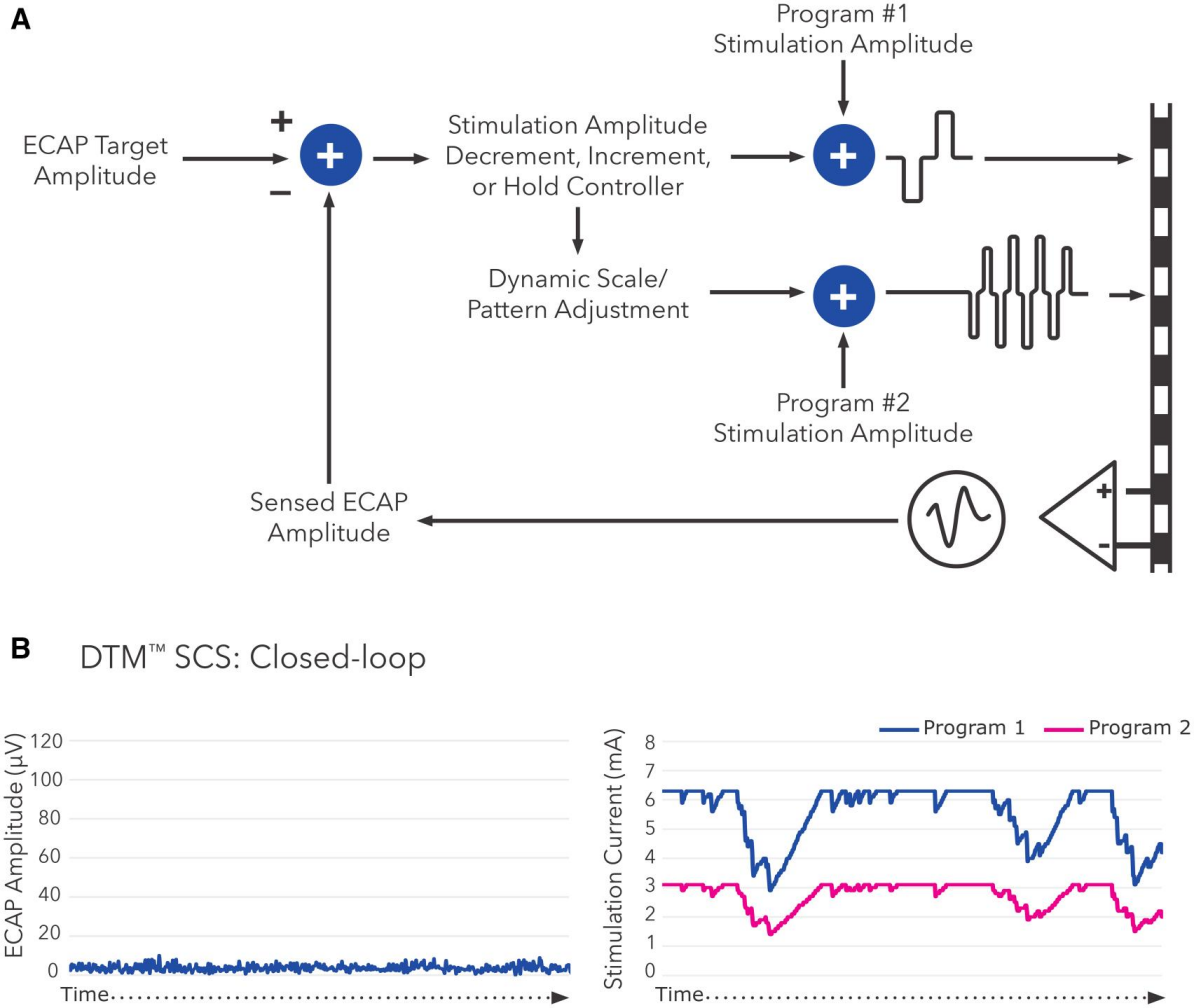
ECAP-controlled CL-SCS with DTM waveforms. (A) ECAPs sensed in response to each pulse from Program #1 are used to determine the next stimulation pulse for CL control of both Programs #1 and #2. The feedback loop controller determines, based on the ECAP, whether stimulation amplitude should hold, decrement, or increment. (B) The ECAP generated during CL-SCS remains near a desired level when viewed over time. When the ECAP reaches an upper threshold limit, CL-SCS decreases the stimulation amplitude of both programs to ensure a more consistent VTA. The 2 stimulation traces increase and decrease proportionally during CL-SCS.

Toward the goal of providing pain relief, SCS patients have access to a range of programming options beyond low-frequency, paresthesia-based SCS therapy. Therefore, CL systems must also consider patients who prefer alternative stimulation programming in which the amplitude is adjusted to near or below the perception threshold for single or multiplexed waveforms. The CL feature available in the Inceptiv device (Medtronic) can adjust conventional or contemporary waveforms, including differential targeted multiplexed (DTM) SCS, programmed above, near, or below perception. Recording accurate ECAPs near the perception threshold amid the noise of other high-frequency stimulation required new ECAP detection and control technology.^34^ These advancements extend the use of ECAPs beyond low-frequency SCS studied in the Evoke trial and allow control of more recently developed stimulation patterns.^10^ For SCS patterns in which low- and high-frequency components are used together, like DTM SCS, the Inceptiv control system uses the ECAP amplitude to modulate both components to provide pulse-to-pulse, physiologically responsive stimulation (Figure 5).^10^ The ECAP is detected following the low-frequency component, but special processing is needed to isolate the ECAP from the electrical noise of both the low- and high-frequency components.

**Figure 6:**
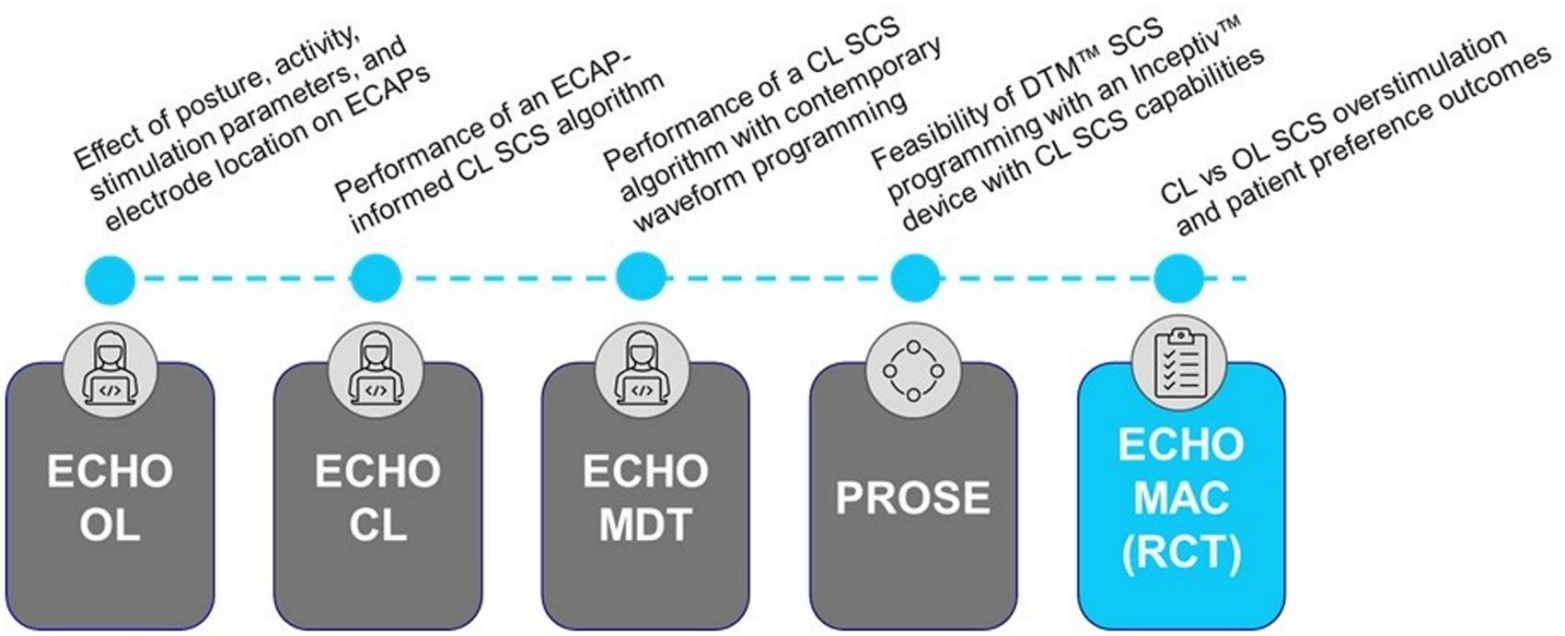
Medtronic closed-loop studies and developmental significance. Studies include the electrically evoked compound action potential human observation (ECHO) open-loop (OL) study,^33^^,^^34^^,^^44^ ECHO closed-loop (CL) study,^45^ ECHO Medtronic (MDT) study,^10^^,^^46–48^ programming optimization for spinal evoked compound action potentials (PROSE),^43^^,^^49^ and the pivotal ECHO Medtronic algorithm comparison (MAC)^42^ randomized controlled trial (RCT).

The clinical development of the ECAP-based CL technology in the Inceptiv neurostimulator began with a series of clinical trials to test and refine the CL algorithm. These initial studies helped to build a foundational understanding of the spinal cord ECAP and the factors that influence ECAP dynamics, and they allowed testing and refinement of the CL algorithm (Figure 6).

The clinical performance of the CL algorithm was tested in a randomized, controlled study (ECHO-MAC) using an investigational device in a clinic setting at the end of the patients’ commercial SCS trial.^42^ Subjects were programmed with a 50 Hz program set near their perception threshold, and the subjects were instructed to perform prescribed movements intended to simulate routine activities of daily living. The participants were randomized and crossed over to OL-SCS or CL-SCS. This in-clinic study, conducted in 42 subjects, found that CL-SCS reduced overstimulation in 97.6% of subjects. Nine out of 10 subjects preferred CL-SCS over OL-SCS.

Additionally, the PROSE study was conducted to develop a workflow to optimize programming of CL-SCS when using DTM SCS therapy.^43^ Most participants in the study had a sufficient ECAP response (39 of 42), and all subjects with a measured ECAP could be programmed with CL and multiplexed waveforms. It took a median of 9 min (range, 2-38 min) to program DTM SCS therapy in the study.^43^

The studies contributing to developing the Inceptiv system all used external investigational hardware, with SCS subjects tested at the end of a scheduled commercial SCS trial. This development pathway helped refine the CL algorithm, demonstrated the benefits of CL in reducing overstimulation and providing consistent therapy, and ensured easy programming of CL when using multiplexed signals.

It is anticipated that even DTM SCS can benefit from CL stimulation control and prevention of overstimulation. As expected in the DTM SCS RCT comparing conventional OL-SCS to OL-DTM SCS, more subjects with conventional SCS experienced temporary uncomfortable stimulation (26.1%) and intense stimulation related to position changes (56.8%).^50^ While most subjects with DTM SCS programming felt no paresthesia during optimal therapy (87%), 28.3% reported intense stimulation related to changing positions.^50^ About 70% of those subjects responded by changing the stimulation intensity or the therapy program. For patients with OL-DTM SCS preferring low amplitude therapy settings at or below perception levels, briefly feeling more intense stimulation (more commonly associated with low-frequency SCS) may cause them to react by decreasing therapy amplitude, leading to chronic understimulation at a suboptimal therapy level.

The CL feature of the Inceptiv device limits the occurrence of overstimulation events, allowing therapy to be programmed at a level closer to perception threshold^51^ without concern that the patient will turn down therapy to avoid overstimulation. For patients desiring therapy at or below their perception level, the ECAP amplitude upper limit—set near perception threshold—can be used as a “smart cruise control” to quickly reduce stimulation amplitude when an ECAP is initially detected (Figure 4B). Once the ECAP amplitude has diminished, stimulation amplitude is ramped back to the set therapy level. Since the initial detection of an ECAP aligns closely with initial patient sensation, reducing stimulation upon ECAP detection prevents the patient from feeling unwanted stimulation. Ultimately, avoiding overstimulation minimizes a patient’s motivation to turn down their therapy, keeping it more consistently at the preset target amplitude and effectively avoiding chronic understimulation.

Using the CL feature with DTM SCS is not just theoretical, but has been investigated in a prospective multicenter trial (NCT05177354).^52^ The main goal of this study was to demonstrate a reduction in overstimulation when applying the CL feature, with additional measurements to characterize pain-related outcomes. Sixty subjects were implanted, and 54 provided data at the 3-month follow-up. During randomized, blinded, in-clinic testing at the 1-month visit, 89.3% of subjects reported a statistically significant reduction in overstimulation.^52^ Preference for CL-SCS was expressed by 86% of subjects. For at-home therapy use, most subjects were programmed to CL-DTM SCS (72%). Other subjects used CL-low dose SCS (11%), different forms of CL SCS (4%), or open-loop programming (13%). Of the 7 (13%) subjects that exhibited a preference for OL-SCS at 3 months, 4 did not have consistent ECAP signals at comfortable stimulation amplitudes, 1 subject had a cardiac pacemaker that interfered with the CL feature, and 2 others used a mix of OL-SCS and CL-SCS. Subjects were set to stimulation parameters deemed comfortable, with mean amplitudes for Program 1 (used to elicit the ECAP signal) at 0.89 (SD: 0.27; *n* = 46) of the perception threshold, and for Program 2 at 0.78 (SD: 0.47; *n* = 46).

At the 3-month follow-up, mean reduction in overall pain was 71.5% (SD: 25.9%). The responder rate (≥50% reduction in pain rating) considering overall pain was 86%. The responder rate was 88.4% for low back pain (*n* = 43) and 100% for upper limb pain (*n* = 3). On the PGIC assessment, 83% reported being a great deal better/better. The study had no unanticipated adverse events, and 7 serious adverse events were reported in 7 participants (9.3%). The study will monitor participants for 2 years. At the 12-month follow-up, 82% of subjects with low back pain (*n* = 38) responded to treatment,^53^ with most continuing to use CL DTM SCS.^53^^,^^54^ While these results are promising, an RCT comparing CL and OL SCS is needed to determine whether CL therapy provides improved pain relief with DTM SCS therapy.

## Limitations and future directions

One limitation of CL technology is that it requires ECAPs to be recordable in order to be useful; consequently, its applicability may be limited, as ECAPs may not be measurable in all patients. It is important to emphasize that CL technology is not a therapy itself, but a mechanism for modulating the dose of therapy delivered to the spinal cord; as such, CL algorithms should be designed for optimal use with any desired waveform, not limited solely to conventional SCS. Notably, ECAPs are not required for patients to benefit from a therapy like DTM SCS, which uses higher frequencies and may be programmed subperception. However, when ECAPs are present, CL technology can enhance the stimulation dose consistency and comfort of stimulation for the patient. Randomized studies have confirmed that CL algorithms control stimulation and minimize overstimulation, supporting this benefit of CL technology.

Regarding the potential for CL to enhance pain relief, it is essential to note that only the EVOKE study has provided RCT data showing improved pain relief when using low-frequency conventional therapy. Open-loop DTM SCS is proven to be more effective for pain relief than conventional SCS; whether CL control with DTM SCS provides further benefit is unknown. The primary advantages of CL DTM SCS may pertain to enhancements in the patient’s therapeutic experience.

Another potential limitation of CL technology is that it does not eliminate the need for reprogramming, as patient preference and comfort level may vary over time. Furthermore, the ECAP is not an indicator of the correct therapy dose for a patient. Optimal programming continues to rely on identifying programming parameters that address the patients’ pain and configuring appropriate ECAP windows or targets. Given this reality, there may be other, yet to be discovered, CL feedback signal(s) that could be used instead of or combined with the ECAP to enhance SCS dose control further. Notably, future feedback signals may differ for paresthesia-based therapies and those programmed near the perception threshold, given the differing mechanisms of these therapies. Finally, as ECAP-based CL technology is still relatively new, the majority of studies conducted have all been industry-sponsored. As the technology becomes more widely adopted, we anticipate the publication of more independent research and further innovation.

## Conclusions

All SCS therapies delivered by fixed-output systems result in inconsistent neural activation due to spinal cord movement, which could affect the long-term use and clinical benefits of SCS therapy. ECAP-controlled CL-SCS technology improves spinal cord dose consistency for many patients who experience under- and overstimulation events. Compared to OL-SCS, patients with CL-SCS systems exhibit a reduced tendency to decrease stimulation amplitudes. Maintaining effective therapy levels may have a positive impact on long-term use and the benefits of the device. While CL technology is an elegant solution to the problem of spinal cord movement, its implementation strategies vary. The methods used to sense the ECAP signal and isolate it from stimulation artifacts impact how the CL algorithm can be used for different SCS therapy preferences. The CL algorithm used in the Evoke system relies on consistent ECAP detection above or near the level of sensation, which makes it well-suited for paresthesia-based therapies. The CL algorithm and filtering methods employed in the Inceptiv neurostimulator were developed to support a broad selection of SCS therapies, including conventional, high-frequency (up to 1200 Hz), and DTM SCS.

## Disclosures

This supplement was sponsored by Medtronic, which provided funding for its publication. No authors received compensation for their contributions to the writing, review, or critical input into the content of this article. A.B., J.G., D.D., and L.J. are Medtronic employees and participated in the manuscript writing and editing. All authors critically reviewed the manuscript and approved the final version.
